# Developmental hip dysplasia treated by total hip arthroplasty using a cementless Wagner cone stem in young adult patients with a small physique

**DOI:** 10.1186/s12891-017-1554-9

**Published:** 2017-05-15

**Authors:** Ping Zhen, Jun Liu, Hao Lu, Hui Chen, Xusheng Li, Shenghu Zhou

**Affiliations:** 1 Department of Orthopedics, The Second Affiliated Hospital of Lanzhou University, Cuiying Door, No. 82, Chengguan District, Lanzhou City, Gansu Province 730030 People’s Republic of China; 2 Department of Orthopaedics, Lanzhou General Hospital of PLA, South Binhe Road, No. 333, Lanzhou City, Gansu Province 730050 People’s Republic of China

**Keywords:** Total hip arthroplasty, Cementless femoral stem, Wagner cone stem, Developmental hip dysplasia, Small physique

## Abstract

**Background:**

Developmental hip dysplasia (DDH) may lead to severe acetabular and femoral abnormalities that can render total hip arthroplasty (THA) challenging, especially in DDH patients with a small physique. Most conventional cemented or cementless femoral components are often difficult to implant in the narrow femoral canal and require slight version correction during surgery. The aim of this study was to present the mid-term results of THA in the treatment of DDH patients with a small physique using a cementless Wagner cone prosthesis (Zimmer®, US).

**Methods:**

Between January 2006 and March 2010, we retrospectively reviewed 50 patients who were treated at our center. A total of 50 patients (52 hips; 45 women, five men; mean age 32.5 years; range 27 to 38 years) who underwent THA were observed. The mean femoral medullary canal dimension at the isthmus was 7.6 mm (range 6.0 to 8.7). According to the Crowe classification, 19 hips presented dysplasia of grade I, while 33 presented dysplasia of grade II. All patients were treated with THA using a cementless Wagner cone prosthesis. Clinical and radiologic evaluations were performed on all patients.

**Results:**

The mean duration of follow-up was 7.7 years (range 5.4 to 10.5). The Harris hip score (HHS) improved from 63 ± 9 (range 55 to 70) pre-operatively to 92 ± 8 (range 88 to 100) at the last follow-up. The HHS at the most recent follow-up was excellent in 66% of patients (34 hips), good in 26% (14 hips), fair in 6% (3 hips), and poor in 2% (1 hip). Radiographic evaluation demonstrated excellent osteointegration of the implants. Stem subsidence was present in three stems, and the range of stem subsidence was 2 mm in two stems (3.9%) and 3 mm in one stem (1.9%). Femoral osteolysis was observed in nine hips (18%) in the proximal zones, and no distal osteolysis was noted. Heterotopic ossification was observed in three hips (5.8%); of these, two were classified as Brooker’s grade 1, and one was classified as Brooker’s grade 2 at the most recent follow-up. None of the implants were revised.

**Conclusions:**

Based on the tapered shape and free setting of anteversion, the Wagner cone femoral stem facilitates its implantation in dysplastic hips. Therefore, this series of short stems with a smaller diameter can ensure safe implantation in narrow medullary canals, especially in young DDH patients with a small physique.

**Trial registration:**

Registration Number: ChiCTR-ORC-17011181.

Reg Date: 2017-04-19 00:44:59

Retrospective registration

## Background

Developmental hip dysplasia (DDH) consists of abnormal development of the hip joint that is characterized by anatomical alterations involving both the acetabulum and the femur [1, 2]. In DDH, significant deformities, such as dysplastic acetabulum, deformity of the femoral head and a shortened femoral neck with excessive anteversion, may be present [2–5]. Especially in some Asian DDH patients with a small physique, the femur usually presents with a short skeleton and an excessive narrow canal [6]. Total hip arthroplasty (THA) is technically difficult in such small-proportioned patients because most conventional cemented or cementless femoral components are often difficult to implant in the narrow femoral canal and require slight version correction of the femoral stem [6–9].

The Wagner stem was originally designed in 1987 [10] to treat patients with severe proximal femoral bone loss in revision surgery [11, 12]. The Wagner cone prosthesis hip stem was designed for uncemented fixation in challenging bone conditions at the proximal femur, such as proximal femoral deformities, especially in DDH patients [1]. The circular cross-section of this implant facilitates unimpeded rotation during implantation and enables the free setting of anteversion to restore normal anteversion in dysplastic hips [7, 8, 13]. Meanwhile, a series of short stems are available in 12 diameters from 6.4 to 15.4 mm to appropriately fit the intramedullary canal in DDH patients with a small physique [14].

Since 2006, we have performed total hip replacements for 52 joints in 50 young adult patients with DDH (Crowe I and II). Forty-eight patients underwent a unilateral procedure, and two underwent a bilateral staged procedure. The aim of this study was to present the clinical and radiological outcomes of THA in the treatment of DDH patients with a small physique by cementless Wagner cone prosthesis in our center after a minimum follow-up of 5.4 years.

## Methods

### Patient demographics

Between January 2006 and March 2010, 50 patients (52 hips) with osteoarthritis secondary to hip dysplasia types I and II according to the Crowe classification underwent THA in our center. The average age of the 45 females and five males at the time of surgery was 32.5 years (27–38). The patients’ mean height, body weight, and body mass index (BMI) were 156 ± 2.5 cm (160 ± 5.6 cm), 50 ± 3.2 kg (53 ± 6.3 kg), and 21.8 ± 3.8 (23.1 ± 2.8), respectively. All operations were performed by one of the members of our institution’s hip team (Ping Zhen or Xusheng Li).

Pre-operatively, all patients were evaluated clinically and radiographically. The Harris hip score (HHS) was used for clinical evaluation. For all patients, standard radiographs of the pelvis in anteroposterior view and of the affected hip in axial view were obtained. On radiographs, the degree of dysplasia was determined using the Crowe system [15], based on which 19 hips presented dysplasia grade I and 33 presented grade II. The transverse diameter of the medullary canal was also measured on an anteroposterior view of the femur using a custom-programmed, semi-automated analysis software module within ImagePro Plus (Mediacybernetics, Silver Springs, MD) [16]. The femoral medullary canal dimensions at the isthmus ranged from 6.0 to 8.7 mm (mean, 7.6 mm) in this study. In addition, computed tomography (CT scans) were performed on 38 patients to evaluate the structure of the dysplastic acetabulum and antetorsion of the femur head. The mean antetorsion angle of the femur head was 52° (range 46° to 62°) in these patients. Based on radiographs, thorough pre-operative planning was conducted for all patients, including assessment of the available bone stock and determination of the position and choice of the femoral and acetabular implants. The appropriate implant and femoral offset were chosen by pre-operative templating. Patient demographics are listed in Table 1.

**Table 1:** Patient demographics

Demographic	Data
Gender	5 male (5 hips), 45 female (47 hips)
Mean age	32.5 years (range 27 to 38)
Mean height	156 ± 2.5 cm (160 ± 5.6 cm)
Mean weight	50 ± 3.2 kg (53 ± 6.3 kg)
BMI	21.8 ± 3.8 (23.1 ± 2.8)
Classification of Crowe	19 hips of grade I, 33 hips of grade II
Mean antetorsion angle	52° (range 46° to 62°)
Mean dimension of the femoral medullary canal (at the isthmus)	7.6 mm (range 6.0 to 8.7 mm)
Stem length	18 stems in 100.5 mm, 34 in 110 mm
Distal diameter of the stem	18 stems in 6.4 mm, 16 in 7.4 mm, 7 in 8.4 mm, 6 in 9.4 mm, 5 in 10.4 mm
Mean follow-up	7.7 years (range 5.4 to 10.5)

### Surgical technique and implantation

THA was performed in patients under general or spinal anesthesia. A posterolateral approach was used, with the patient placed on the contralateral side. After capsulotomy, we confirmed the position of the femoral head in relation to the acetabulum. The hip was then dislocated, and osteotomy of the femoral neck was performed at the designated level. The primitive acetabulum was reamed, and an uncemented, press-fit cup was applied in or close to the anatomical position. A box osteotome, trochanteric reamer or a burr was used to remove bone from the medial portion of the greater trochanter and the lateral portion of the femoral neck. The femoral medullary canal was progressively reamed with tapered reamers in the longitudinal direction of the femur until noticeable resistance was felt. A Wagner cone prosthesis uncemented stem was inserted by guiding it with its appropriate device, aiming to obtain approximately 10–20° of anteversion. The prosthesis was rotated into the desired anteversion and was impacted into its definitive position with a few moderate mallet blows. The correct depth of penetration as per the pre-operative planning was verified. After the hip was reduced, the stability and range of motion were assessed, and the appropriate limb length was verified in comparison to the contralateral limb. In this series of patients, a ceramic-polyethylene bearing was used in 27 hips, and a ceramic-ceramic bearing was used in 25 hips.

**Fig. 1:**
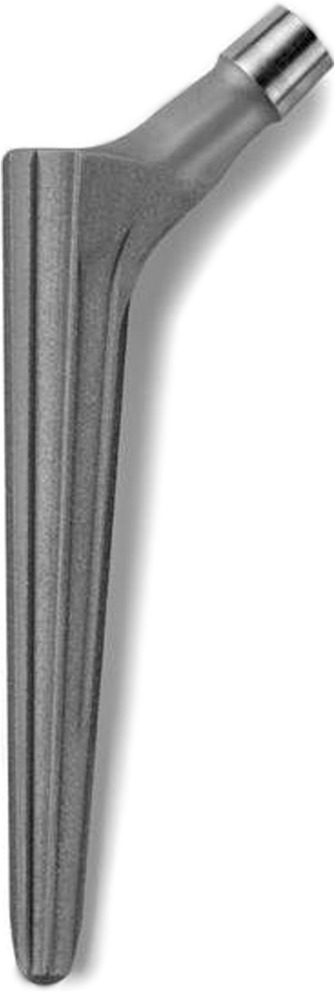
The Wagner cone prosthesis has a tapered shape with a cone angle of 5°, and the 8 sharp longitudinal ribs of the stem are beneficial for bony apposition and optimum rotational stability

**Fig. 2:**
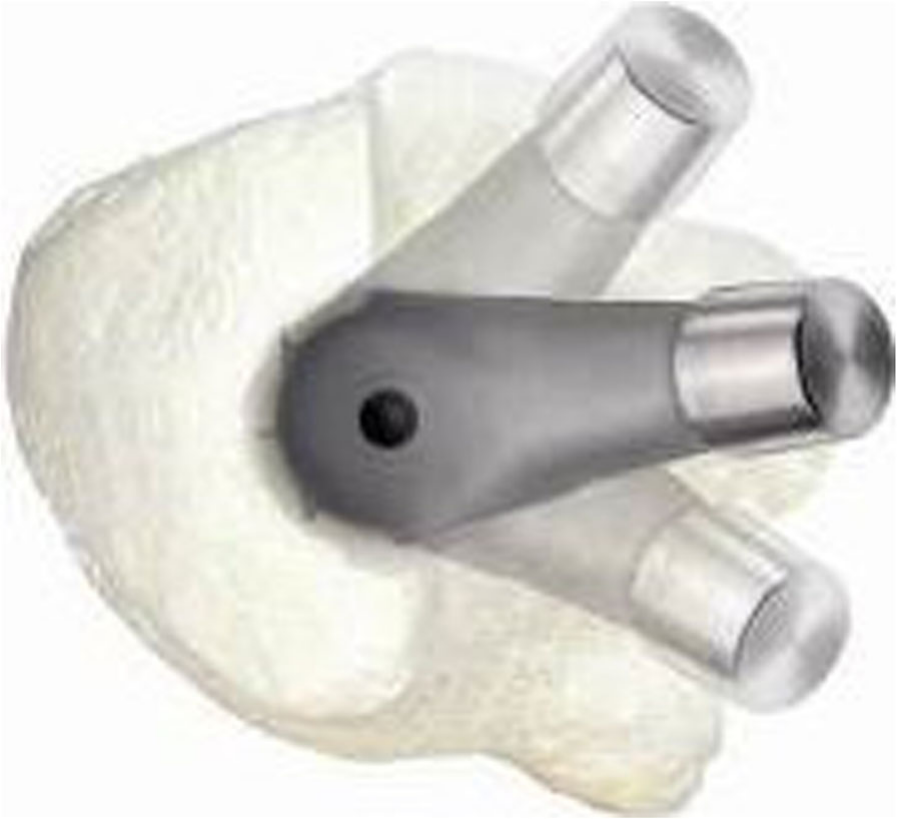
The circular cross-section facilitates unimpeded rotation during implantation to enable the free setting of anteversion

Considering that the main problem regarding the femur, as noted previously, is excessive anteversion of the femoral neck and the small femoral canal in DDH [7], the use of the uncemented Wagner cone prosthesis hip stem (Zimmer ®, US) allowed us to correct the femoral head version and to ensure safe implantation of the femoral stem. Based on a 5° tapered stem with a circular cross-section (Fig. 1), the Wagner cone prosthesis stem can be placed in any anteversion orientation by the surgeon (Fig. 2). The new Wagner cone prosthesis stem is available in two different neck angles (125° and 135°) and provides good options to facilitate appropriate restoration of biomechanical parameters, including the center of rotation, offset and leg length. This series of cone stems is available in lengths from 100.5 to 110 mm and in diameters from 6.4 to 10.4 mm (distal section distance: at 96 mm distance from the shoulder of the prosthesis). In this study, in 18 THAs, the inserted femoral cone stem had a diameter of 6.4 mm; in 16 cases, the stem diameter was 7.4 mm; in seven cases, the diameter was 8.4 mm; in six cases, the diameter was 9.4 mm; and in five cases, the diameter was 10.4 mm.

### Follow-up and radiographic analysis

Clinical and radiographic follow-up were performed every 2 years postoperatively. Clinical evaluation was carried out using the HHS. Routine radiographs comprising an anteroposterior view of the pelvis as well as anteroposterior and lateral views of the affected hip were performed and compared to the initial postoperative radiographs. Radiographs were evaluated by two fellowship-trained hip arthroplasty surgeons (MD and NR) for evidence of biological fixation (using the fixation/stability score of Engh [17]), subsidence (as described by Pelligrini et al [18]), osteolysis (based on Gruen modes of failure [19]), and heterotopic ossification (graded according to Brooker’s classification [20]).

## Results

The mean duration of follow-up was 7.7 years (range 5.4–10.5 years). Two patients with two THAs were lost to follow-up, one after six and the other after 8 years.

### Clinical outcome

The HHS improved between the pre-operative evaluation and the last follow-up in this group. The average HHS was 63 ± 9 (range 55–70) pre-operatively, 90 ± 7 (range 82–98) 12 months after surgery, and 92 ± 8 (range 88–100) at the final follow-up. The HHS at the most recent follow-up was excellent in 66% of the patients (34 hips), good in 26% (14 hips), fair in 6% (3 hips), and poor in 2% (1 hip). Twenty-eight patients (30 hips) had no limp (60%), 16 patients (16 hips) had a mild limp (32%), three patients (3 hips, 6%) had a moderate limp, and one patient (1 hip) had a severe limp (2%). Mild thigh pain was described in association with three hips (6%) within 1 year; this pain typically occurred only with prolonged activity, was not disabling, and did not require medication. One patient reported mild pain in the groin. The mean correction of limb length (comparing the pre-operative length with that at the last radiological follow-up) was 2.2 cm (range 1.5 to 3.2 cm). The mean pre-operative and postoperative discrepancies were 2.29 ± 1.05 cm and 0.67 ± 1.13 cm, respectively.

### Radiographic assessment of the femoral component

**Fig. 3:**
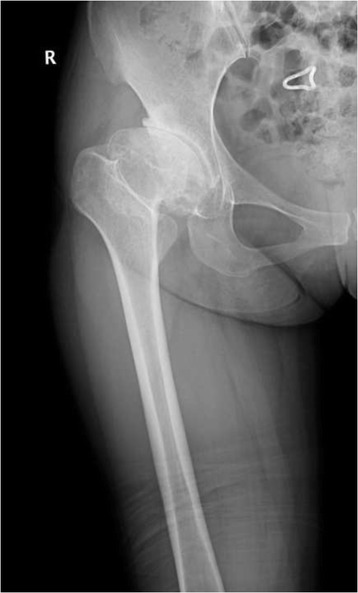
Pre-operative radiographic aspect in an anteroposterior view of a 32-year-old female with Crowe grade II developmental hip dysplasia on her right side. The patient presented with a narrow femoral canal and excessive anteversion of the femoral neck

**Fig. 4:**
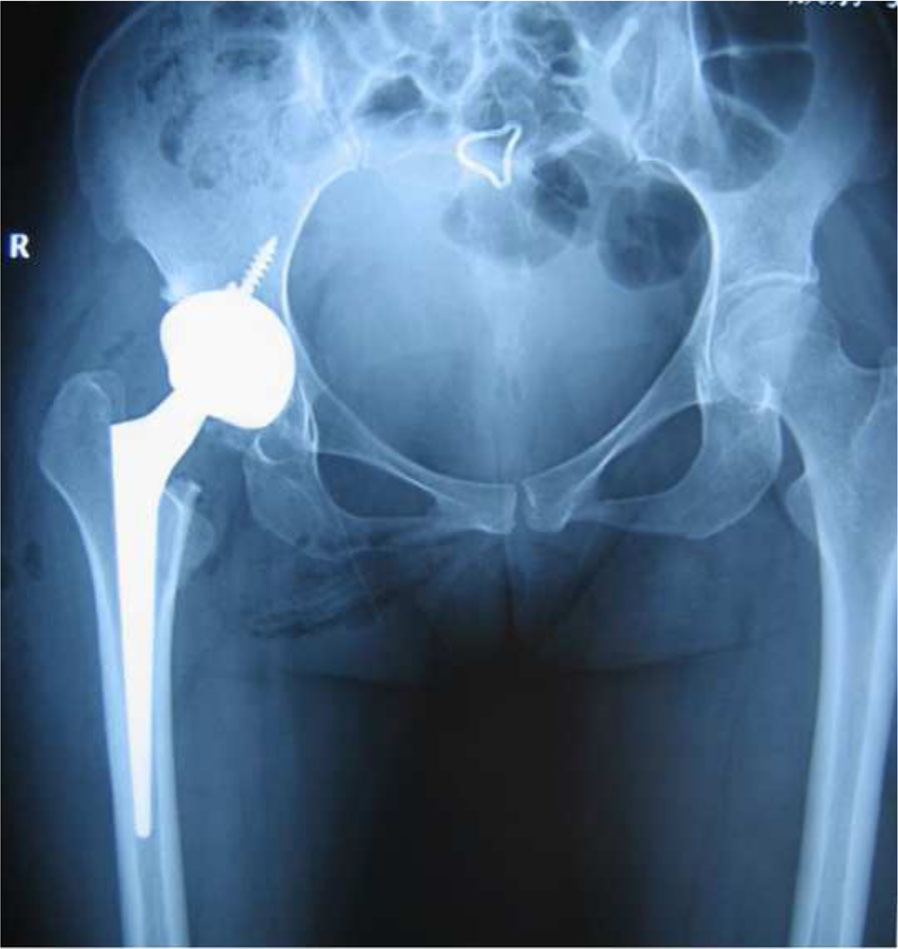
Immediate postoperative anteroposterior radiographs demonstrating correct placement of the implant and normal anteversion

**Fig. 5:**
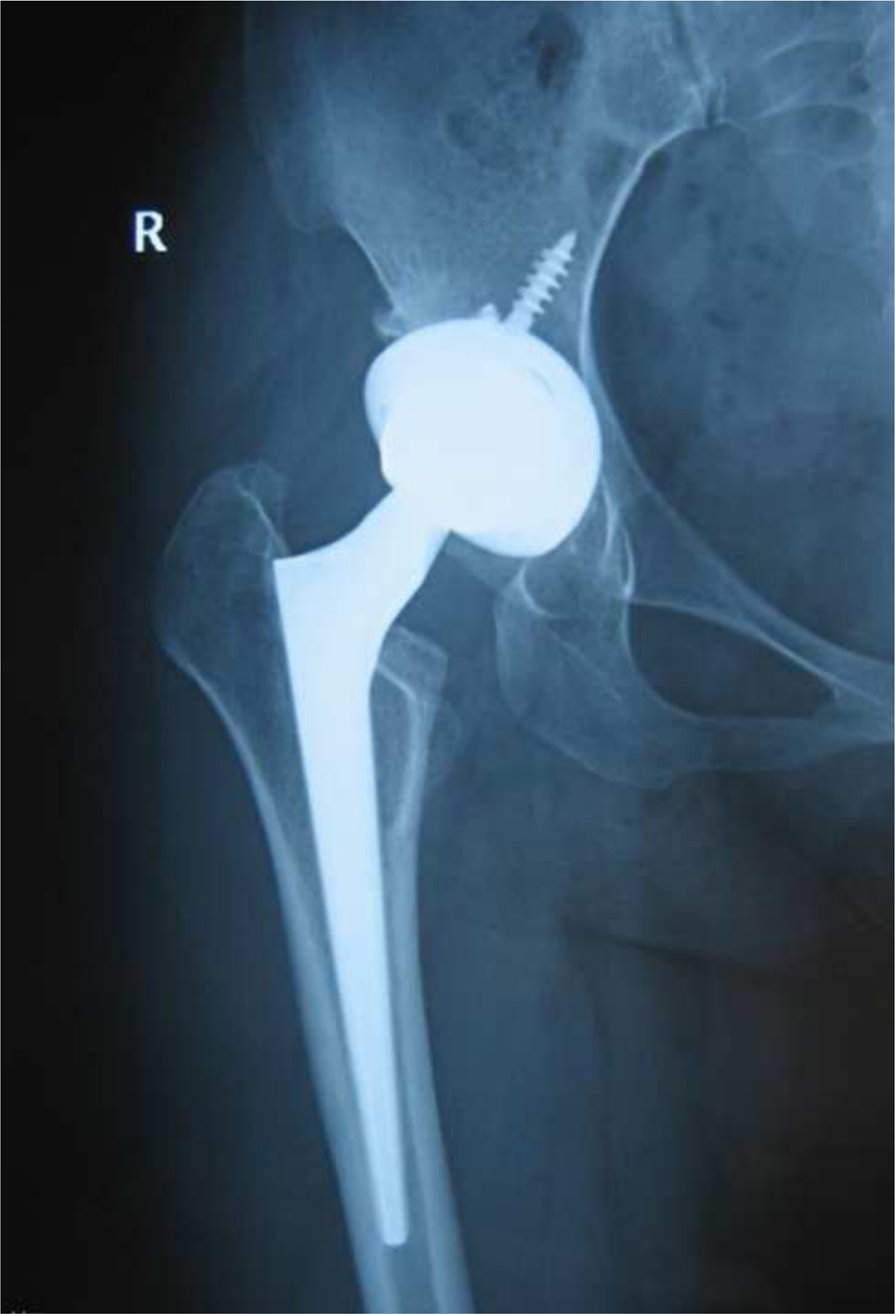
Radiographic aspect in the anteroposterior view at 8 years of follow-up: the implant is stable, and no signs of bone resorption are noticeable

**Fig. 6:**
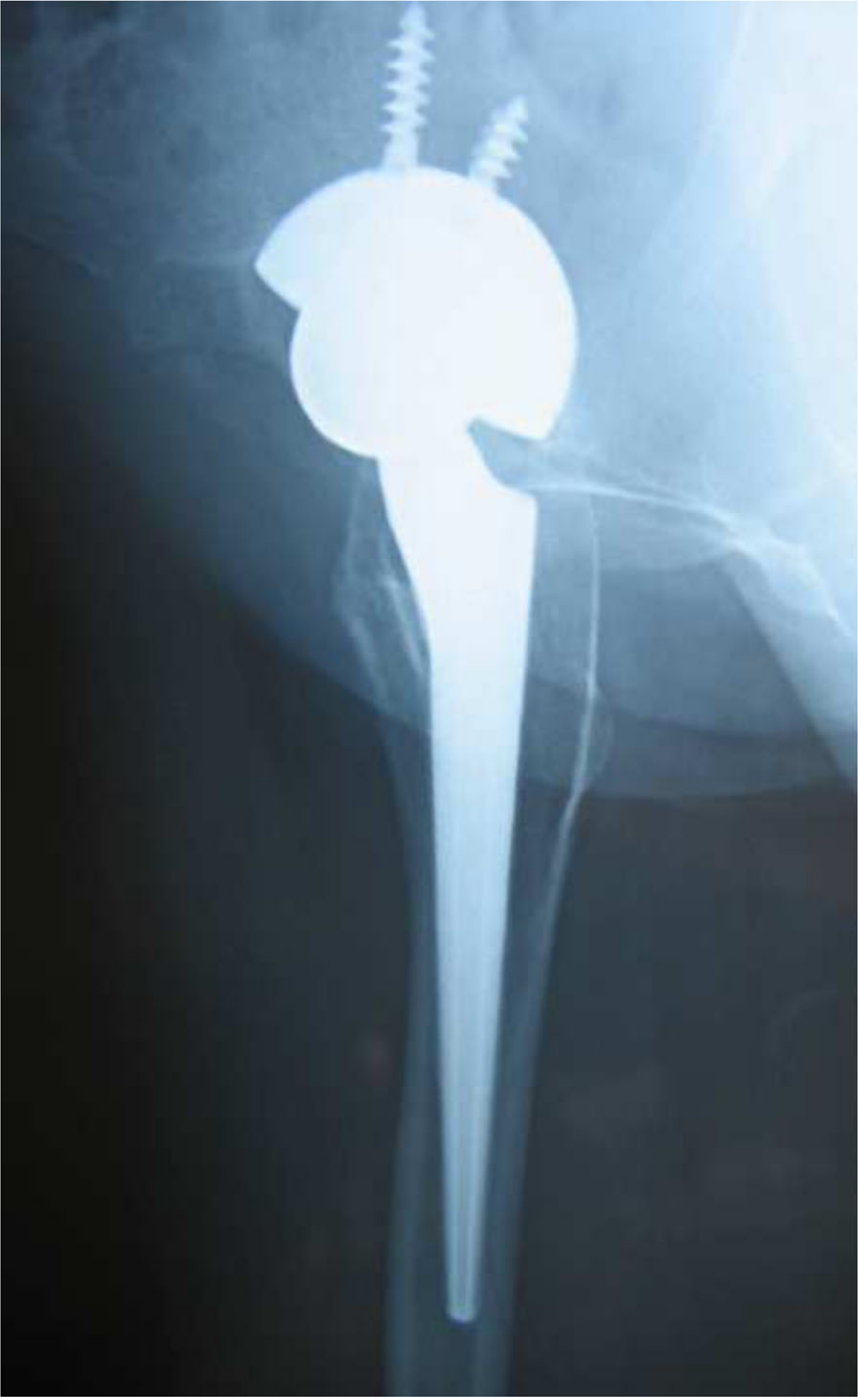
Frog-leg lateral radiograph on the right side at 8 years of follow-up

**Fig. 7:**
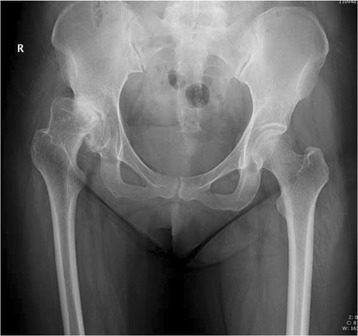
Pre-operative radiographic aspect in an anteroposterior view of a 40-year-old female with Crowe grade II hip dysplasia on her right side. The patient presented with a narrow femoral canal

**Fig. 8:**
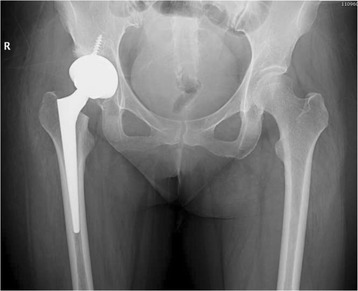
Immediate postoperative anteroposterior radiographs demonstrating correct placement of the implant. Press-fit of the stem was achieved

**Fig. 9:**
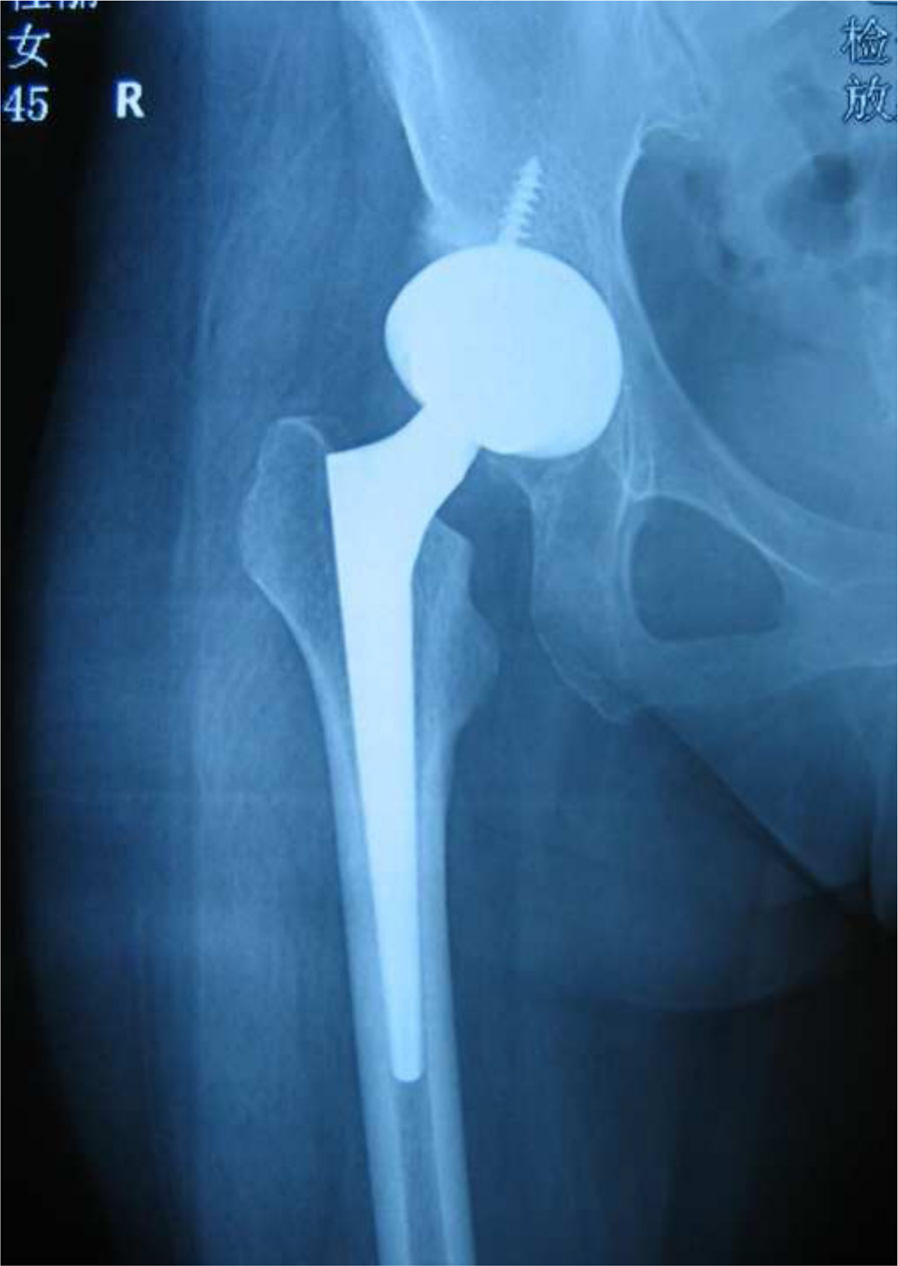
Radiographic aspect in the anteroposterior view at 5 years of follow-up: the implant is stable, and no signs of bone resorption are noticeable

Press-fit of the stem was achieved in 49 joints (94.2%) immediately after surgery. Three stems were shown to be slightly undersized immediately after surgery. Anatomical reconstruction of the correct antetorsion was achieved in all cases (Figs. 3, 4, 5 and 6). Based on the Engh classification, stable osseous ingrowth was confirmed in 48 joint stems (92.3%) and was suspected in two stems (3.9%); in the remaining two stems (3.9%), stable fibrous fixation was evident. There were no unstable stems (Figs. 7, 8 and 9). Stem subsidence was present in three stems; the stem subsidence ranged from 2 mm, in two stems (3.9%), to 3 mm, in one stem (1.9%). Nonprogressive radiolucent lines were found in Gruen’s zone 1 in nine patients, in zone 7 in one patient, in zones 1 and 7 in seven patients, in zones 1, 2, and 7 in one patient, and in zones 1, 2, 6, and 7 in one patient. Femoral osteolysis was observed in nine hips (18%) in the proximal zones (zone 1 and 7 in five hips, zone 1 alone in three hips, and zone 7 alone in one hip). No distal osteolysis was noted. No femoral prosthesis developed varus or a valgus tilt over time. Heterotopic ossification was observed in three hips (5.8%): of these, two showed Brooker’s grade 1 and one showed Brooker’s grade 2 at the most recent follow-up. No stem revisions occurred in this study.

There were no cases of intraoperative femoral fracture or femoral perforation during rasping of the canal or stem implantation, nor did deep infections or wound-healing problems occur postoperatively. One dislocation developed within 1 month after the procedure. This patient was treated with a closed reduction and a hip brace without the need for further surgery. No recurrence was observed.

## Discussion

DDH routinely presents as hip incongruence secondary to low or high dislocation. The associated anatomical deformities of the hip include hypoplastic acetabulum and deformity of the proximal femur [1–3, 7–9]. If not diagnosed and adequately treated during childhood, DDH may lead to early functional impairment and anatomical alterations of the hip joint in young adults [1]. Many surgical strategies, including acetabular reconstruction, femoral osteotomy and hip arthroplasty, have been proposed for the treatment of DDH in adolescents [1]. Based on good-to-excellent hip-joint congruency in joint-preserving surgery, periacetabular and femoral osteotomies have been recommended if degeneration of the articular cartilage is minimal [8], especially in patients <30 years of age [21].

As a progressive treatment, THA in young DDH patients remains widely debated [7–9] because the young age of the patient raises the issue of the long-term survival of the implant [1, 22, 23]. However, THA is the optimum therapy in such patients with severe subluxation or late coxarthrosis [22–24].

DDH may lead to severe acetabular and femoral abnormalities, including small acetabular and femoral dimensions, soft tissue contracture, inadequate acetabular bone stock, and excessive femoral anteversion [1, 3, 7–9]. These anatomical alterations increase the difficulty of THA and may also threaten the long-term survival of the prosthetic implant [1, 6–9, 25–27]. The main problems associated with the femur are a deformed head, a short neck with excessive anteversion and a narrow femoral canal [2, 3]. DDH patients can have small physiques [25–28], especially in Asian patients [6]. Some narrow femoral canals are too small to accommodate many standard femoral components during THA [25]. In early studies, the treatment of hip disease in such patients often required the use of miniature or micro-miniature femoral components, and a cemented technique was preferred [25, 28]; however, significant problems have been encountered in these situations, including intraoperative femoral fracture, instability, poor femoral cement mantle and component loosening due to the smaller femoral anatomy [25]. Meanwhile, the young age of DDH patients increases the importance of the long-term survival of the cemented implants [1]. Although no precise definition of a small physique and narrow femoral canal have been developed in previous studies [6, 25–28], most cementless standard stems in small-proportioned patients are also limited [26] because current cementless stems are designed to extend to the isthmus of the medullary canal to stabilize component alignment and prevent varus migration [7]. When such patients undergo hip arthroplasty, standard cementless femoral stems always encounter this difficulty in insertion, and extensive reaming and rasping are used to obtain safe implantation [29]; however, more vigorous reaming may easily cause perforation or fracture during the operation. Meanwhile, dysplastic hips usually present with excessive anteversion of the femoral head, and anatomical reconstruction of the correct antetorsion should be achieved by rotating the stem in the medullary canal. However, most conventional cementless stems that are designed with rectangular cross-sections generally fill the metaphysis of the proximal femur. Consequently, there is little opportunity to alter the stem version after insertion [26, 30–32]. Thus, for this abnormality in proximal femoral anatomy, an alternative to the use of a nonmodular femoral component is often needed [26]. Modular implants, such as the S-ROM prosthesis [26, 32] and custom-made prostheses [33], allow for variable versions, thus providing the ability to alter the version to help balance the hip with respect to internal and external rotation [2, 6, 10–12]. Moreover, S-ROM cementless femoral stems are available in a variety of distal diameters from 9 to 23 mm (in Japan, S-ROM stems as small as 6 mm are available) [26] that can fit in small femoral canals. In fact, modular prostheses are always accompanied by a subtrochanteric shorting osteotomy procedure for hips with high dislocation [26, 32, 34] or an atypical femoral geometry [34]. Moreover, modular femoral components also raise concerns about fretting, corrosion, and mechanical failure [35, 36], and an increasing number of reports have described the failure of modular implants due to fracture at the modular junctions [37, 38].

Although several prostheses have been used for treating DDH [8, 26, 28, 29, 31–33], only a few studies have considered the Wagner stem as a possibility [1, 7, 13, 30]. Based on its 5° tapered stem with a circular cross-section, the Wagner cone prosthesis stem can be placed in any anteversion orientation by the surgeon. Moreover, as a relatively short stem with a small diameter, the Wagner cone prosthesis can easily be implanted in narrow medullary canals, comparable to conventional cemented or uncemented stems [1, 7, 14, 39, 40]. The conical stem used in these cases allows good correction of the over-anteverted neck, and the eight-rib design provides good rotational stability, In this study, indications for the Wagner cone prosthesis are femurs with slim and narrow canals (medullary canal diameter at isthmus <10 mm) or combined with excessive anteversion of the neck (femoral anteversion of >40°) in young DDH patients, in which the femoral shortening osteotomy is not needed in Crowe type I and II dysplastic hips. In this series, excellent survival of the stem was accomplished (a mean of 7.7 years), and minimal complications were observed. Despite the small size of the titanium alloy femoral components, no bending or fracture of the femoral stem was observed. Thus, this cone prosthesis is a good and inexpensive solution for cases of Crowe type I and II dysplastic hips.

This study should be interpreted in light of several important limitations. First, this study did not compare the results of THA using Wagner cone stems and some standard cementless stems. However, by comparing the present cohort with our historical control, we concluded that the Wagner-based procedure resulted in satisfactory outcomes. Second, this was a retrospective study with all the problems inherent with this methodology. Third, this study was unable to separately analyze bilateral and unilateral cases.

## Conclusion

Based on its free setting of anteversion and small diameter, the new Wagner cone femoral stem facilitates its implantation in dysplastic hips with narrow femoral canals and excessive anteversion. Further studies aimed at better defining the indications for this procedure will lead to even better results. Further follow-up is also needed to confirm whether these results will be maintained over a longer term.

## Abbreviations

BMI: Body mass index
THA: Total hip arthroplasty
